# Supplementary material for: SBpipe: a collection of pipelines for automating repetitive simulation and analysis tasks
Source: BMC Syst Biol. 2017 Apr 10;11:46. doi: 10.1186/s12918-017-0423-3 (PMC5387271; doi:10.1186/s12918-017-0423-3)
Supplement: Additional file 1 — Supporting information. Additional file containing supporting Tables S1–S4 and Figures S1–S15. (PDF 29,712 kb) [file 12918_2017_423_MOESM1_ESM.pdf]

Supporting Information for SBpipe: a collection of pipelines for systems modelling of biological networks

Piero Dalle Pezze*[1] and Nicolas Le Novère[1]

[1]The Babraham Institute, Babraham Campus, CB22 3AT, Cambridge, UK

*Correspondence: piero.dallepezze@babraham.ac.uk

List of Tables

S1 Computational model equations and parameters . . . 3
S2 Data sets used for parameter estimation . . . 4
S3 Parameter estimation results . . . 5
S4 Parameter estimation results with identifiability issues . . . 6

List of Figures

S1 Diagram for the insulin receptor test model . . . 7
S2 Example of configuration file for the parameter estimation pipeline . . . 8
S3 Parameter estimation pipeline (1st group) . . . 9
S4 Parameter estimation pipeline (2nd group) . . . 10
S5 Parameter estimation pipeline (3rd group) . . . 11
S6 Parameter estimation pipeline with identifiability issues (1st group) . . . 12
S7 Parameter estimation pipeline with identifiability issues (2nd group) . . . 13
S8 Parameter estimation pipeline with identifiability issues (3rd group) . . . 14
S9 Example of configuration file for the simulate pipeline . . . 15
S10 Simulate pipeline . . . 16
S11 Example of configuration file for the single parameter scan pipeline . . . 17
S12 Single parameter scan pipeline . . . 18
S13 Example of configuration file for the double parameter scan pipeline . . . 19
S14 Double parameter scan pipeline (1st group) . . . 20
S15 Double parameter scan pipeline (2nd group) . . . 21

ODEs:

$$
\begin{aligned}
d\ IR\beta/dt &= -k1 * IR\beta * Insulin + k3 * IR\beta\_refractory \\
d\ IR\beta\_pY1146/dt &= +k1 * IR\beta * Insulin - k2 * IR\beta\_pY1146 \\
d\ IR\beta\_refractory/dt &= +k2 * IR\beta\_pY1146 - k3 * IR\beta\_refractory
\end{aligned}
$$

Parameters:

$$
\begin{aligned}
k1 &= 0.475519 \\
k2 &= 0.471947 \\
k3 &= 0.0578119
\end{aligned}
$$

Initial protein amounts:

$$
\begin{aligned}
Insulin &= 1 \\
IR\beta &= 16.5607 \\
IR\beta\_pY1146 &= 0 \\
IR\beta\_refractory &= 0
\end{aligned}
$$

Table S1: Computational model equations and parameters. A minimal model representing the phosphorylation of the insulin receptor by insulin was used for testing the SB pipe package. *Insulin* is a constant input in the system. For this simple model the cell volume is set to 1. Units of parameters and amounts are arbitrary (a.u.). For a more descriptive graphic representation, see Supplementary Figure S1. This model and the analyses illustrated in this document are available with SBpipe in `$SBPIPE/tests/insulin_receptor`.

Time [min]	$IR\beta\_pY1146$ [a.u.]
0	0
1	3.11
3	3.13
5	2.48
10	1.42
15	1.36
20	1.13
30	1.45
45	0.67
60	0.61
120	0.52
0	0
1	5.58
3	4.41
5	2.09
10	2.08
15	1.81
20	1.26
30	0.75
45	1.56
60	2.32
120	1.94
0	0
1	6.28
3	9.54
5	7.83
10	2.7
15	3.23
20	2.05
30	2.34
45	2.32
60	1.51
120	2.23

(A)

Time [min]	$IR\beta\_pY1146$ [a.u.]
0	0
1	3.11
30	1.45
120	0.52
0	0
1	5.58
30	0.75
120	1.94
0	0
1	6.28
30	2.34
120	2.23

(B)

Table S2: Data sets used for parameter estimation. Two data sets are independently used for parametrising the insulin receptor model. The first data set (A) contains a sufficient amount of data for effective parameter estimation (see Supplementary Table S3 and Supplementary Figures S3–S5). On the contrary, the second data set (B), which is a subset of the first data set, is not sufficient to estimate accurately the parameters (see Supplementary Table S4 and Supplementary Figures S6–S8).

**Parameter**	**Value**	**LeftCI66**	**RightCI66**	**LeftCI95**	**RightCI95**	**LeftCI99**	**RightCI99**
k1	0.475553	0.321811	0.701416	0.251119	0.843858	0.211082	0.988792
k2	0.471932	0.364231	0.644191	0.292821	0.792774	0.268067	0.953182
k3	0.0578121	0.0295403	0.101345	0.0186413	0.149327	0.011239	0.188559

**Parameter**	$\frac{Value}{LeftCI66}$	$\frac{RightCI66}{Value}$	$\frac{Value}{LeftCI95}$	$\frac{RightCI95}{Value}$	$\frac{Value}{LeftCI99}$	$\frac{RightCI99}{Value}$
k1	1.47774	1.474948	1.893736	1.774477	2.25293	2.079247
k2	1.295694	1.365008	1.611674	1.679848	1.7605	2.019744
k3	1.957059	1.753007	3.101291	2.582971	5.143883	3.261584

(A)

**MinObjVal**	**AIC**	**AICc**	**BIC**	**Params**	**DataPoints**	**CL66 ObjVal**	**CL66Fits**	**CL95 ObjVal**	**CL95Fits**	**CL99 ObjVal**	**CL99Fits**
59.208	65.208	66.036	69.698	3	33	66.258	7657	76.511	8046	85.910	8237

(B)

Table S3: Parameter estimation results using the data set in Supplementary Table S2A. A sequence of 250 fits using COPASI's Particle Swarm algorithm was generated in order to estimate the kinetic rate constant parameters $k1$-$k3$. (A) Parameter values and confidence intervals within 66%, 95%, or 99% confidence levels are reported. The ratios between parameter value and left/right confidences are also calculated as a measure of the number of folds between values and their confidence intervals. This table is saved in a file called `param_estim_details.csv` within `Results/MODEL_NAME`. (B) Parameter estimation summary showing the minimum objective value, the associated Akaike information criterion (AIC), the AIC with the corrected term (AICc), the Bayesian information criterion (BIC), the number of estimated parameters, the number of total data points, the objective value for each confidence level, the number of fits below each confidence level. In the calculation of AIC, AICc, and BIC, additive Gaussian measurement noise of width 1 is assumed. If the objective function is χ^2, the term $-lnL(\Theta|y)$ in the information criteria approximates the calculated χ^2. Approximate $100(1-\alpha)\%$ confidence regions for a parameter p are computed by: $\chi^2(p) \leq \chi^2(\hat{p})(1 + \frac{m}{n-m}F^{\alpha}_{m,n-m})$, where $\hat{p}$ are estimations for the parameter p, $F^{\alpha}_{m,n-m}$ is the F-ratio distribution, m is the number of model parameters, n is the number of data points, and α is the significance level. This table is saved in a file called `param_estim_summary.csv` within `Results/MODEL_NAME`. Detailed result plots are shown in Supplementary Figures S3–S5.

Parameter	Value	LeftCI66	RightCI66	LeftCI95	RightCI95	LeftCI99	RightCI99
k1	0.452655	0.307796	+inf	0.258413	+inf	0.199757	+inf
k2	0.362462	0.0899081	2.36685	0.0726681	3.03883	0.0642926	4.11522
k3	0.0404414	0.00597852	0.383936	-inf	0.654436	-inf	1.32428

Parameter	$\frac{Value}{LeftCI66}$	$\frac{RightCI66}{Value}$	$\frac{Value}{LeftCI95}$	$\frac{RightCI95}{Value}$	$\frac{Value}{LeftCI99}$	$\frac{RightCI99}{Value}$
k1	1.470633	+inf	1.751673	+inf	2.266028	+inf
k2	4.031472	6.529926	4.987911	8.383858	5.637694	11.353521
k3	6.76445	9.493638	-inf	16.182328	-inf	32.745652

(A)

MinObjVal	AIC	AICc	BIC	Params	DataPoints	CL66 ObjVal	CL66Fits	CL95 ObjVal	CL95Fits	CL99 ObjVal	CL99Fits
8.49528	14.495	17.495	15.950	3	12	12.206	5961	19.433	6260	28.295	6549

(B)

Table S4: Parameter estimation results with identifiability issues using the data set in Supplementary Table S2B. The confidence intervals which could not be clearly identified are labelled *inf* (infinite) and marked in red. Detailed result plots are shown in Supplementary Figures S6–S8. For more detail, see Supplementary Table S3.

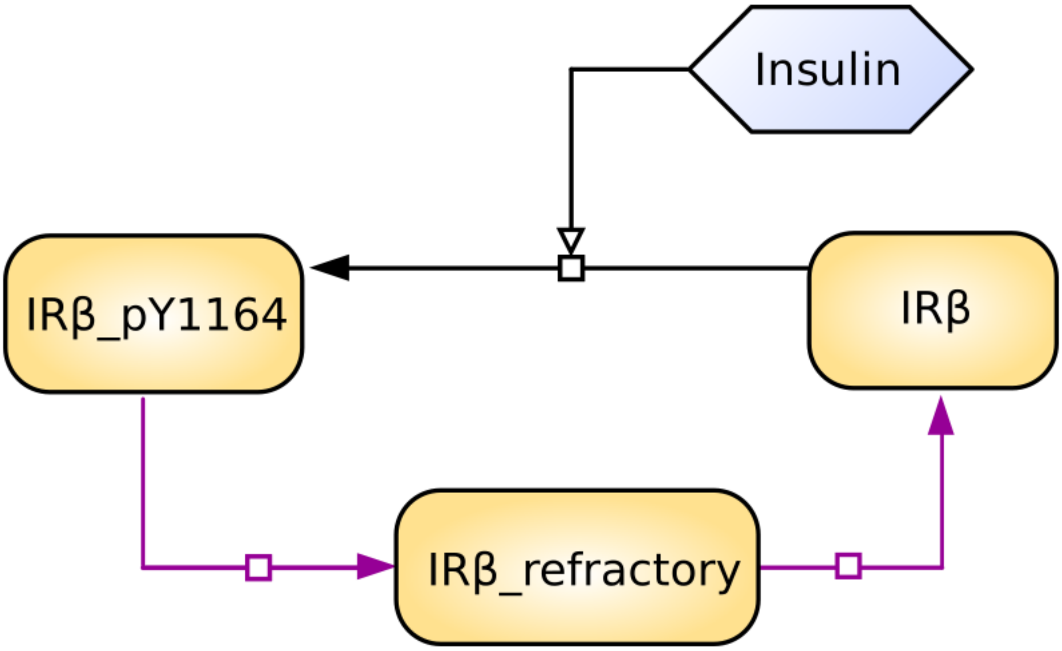

Figure S1: Diagram for the insulin receptor test model. This simplified model of insulin receptor was used for testing the pipeline. In the presence of insulin, the insulin receptor beta ($IR\beta$) is phosphorylated at its Y1164 phosphorylation site. The phosphorylated receptor is then de-phosphorylated and enters in a refractory state. This latter state is used to represent a delay in the system and simplifies the processes of receptor internalisation, degradation and synthesis, reducing the number of model parameters. Finally from this refractory state the receptor can newly become functional.

```
# True if data should be generated, False otherwise
generate_data: True
# True if data should be analysed, False otherwise
analyse_data: True
# True if a report should be generated, False otherwise
generate_report: True
# True if a zipped tarball should be generated, False otherwise
generate_tarball: True
# The relative path to the project directory
project_dir: "."
# The name of the configurator (e.g. Copasi)
simulator: "Copasi"
# The model name
model: "insulin_receptor_param_estim.cps"
# The cluster type. local if the model is run locally,
# sge/lsf if run on cluster.
cluster: "local"
# The number of CPU if local is used, ignored otherwise
local_cpus: 7
# The parameter estimation round which is used to distinguish
# phases of parameter estimations when parameters cannot be
# estimated at the same time
round: 1
# The number of parameter estimations
# (the length of the fit sequence)
runs: 250
# The threshold percentage of the best fits to consider
best_fits_percent: 75
# The number of available data points
data_point_num: 33
# True if 2D all fits plots for 66% confidence levels
# should be plotted. This can be computationally expensive.
plot_2d_66cl_corr: True
# True if 2D all fits plots for 95% confidence levels
# should be plotted. This can be computationally expensive.
plot_2d_95cl_corr: True
# True if 2D all fits plots for 99% confidence levels
# should be plotted. This can be computationally expensive.
plot_2d_99cl_corr: True
# True if parameter values should be plotted in log space.
logspace: True
# True if plot axis labels should be plotted in scientific notation.
scientific_notation: True
```

Figure S2: Example of configuration file for the parameter estimation pipeline. This configuration file was used to generate the Supplementary Table S3 and Supplementary Figures S3–S5. A similar configuration file updating the data fields:
`model: "insulin_receptor_non_identif_param_estim.cps"`
`data_point_num: 12`
was used to generate the Supplementary Table S4 and Supplementary Figures S6–S8. Comments are marked in blue.

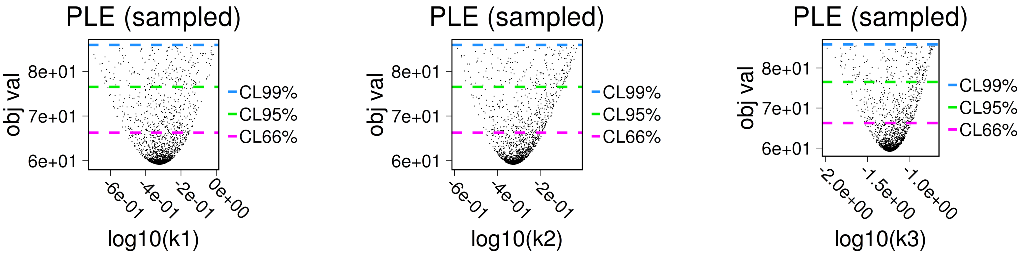

(A)

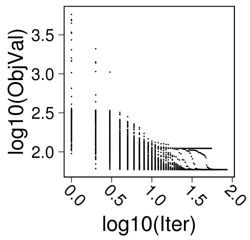

(B)

Figure S3: Parameter estimation pipeline (1st group). Data generated using the configuration file described in Supplementary Figure S2. Parameter estimations were executed using Particle Swarm algorithm in COPASI. (A) Approximated profile likelihood estimations (PLE) for the kinetic rate constants ($k1$-$k3$) are shown. Rather than considering only the parameter sets with the minimum objective value (the best fits calculated by a parameter estimation algorithm), all the parameter sets with objective value lesser than the calculated 99% confidence levels are plotted as black dots. The use of these additional parameter sets allows to sample a portion of the parameter space. For reasonably long fit sequences, these parameter sets can be used to describe the neighbourhood of each estimated parameter. In particular, they can show whether well defined confidence intervals can be calculated for each parameter. Using data set shown in Table S2A, for each of the estimated parameters, parameter sets at the left and right of each minimum value sharply decrease the model fit with the data, suggesting that the parameters are uniquely identifiable. Horizontal lines indicate the confidence levels of 66% (magenta), 95% (green), and 99% (blue), respectively. The estimated parameter values and the respective confidence intervals are reported in Supplementary Table S3. (B) The increase in fit quality with respect to the number of internal iterations performed by a parameter estimation algorithm.

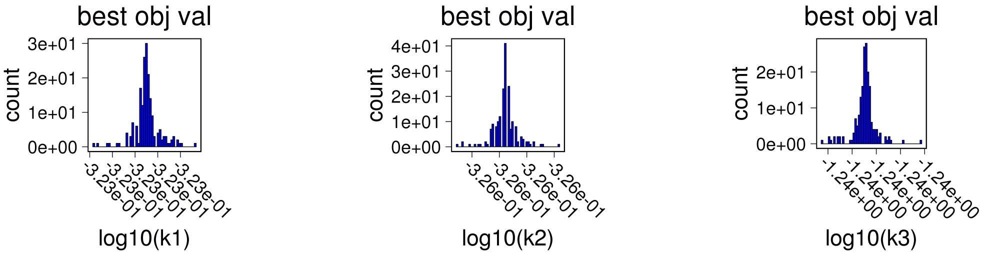

(A)

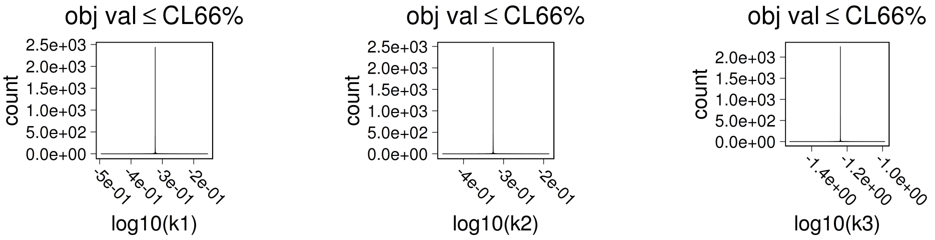

(B)

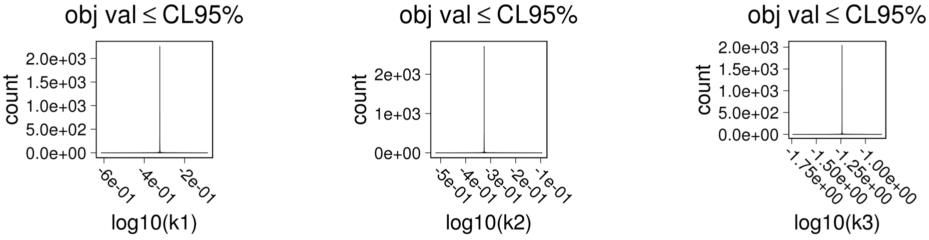

(C)

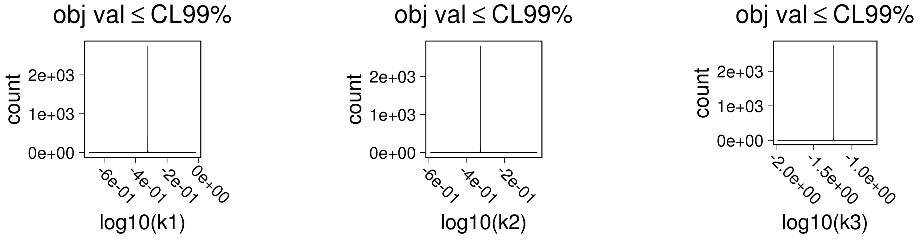

(D)

Figure S4: Parameter estimation pipeline (2nd group). Data generated using the configuration file described in Supplementary Figure S2. This figure shows the distribution of each parameter (column). The number of samples is determined by the applied filter. The first row only includes the best fits (A), whereas the remaining rows include all the fits within the confidence levels of 66% (B), 95% (C), and 99% (D), respectively, as calculated in Supplementary Figure S3. As in this case the parameters are clearly identifiable, these distributions show that all the parameters converge to single parameter values (the parameter optima). For non-identifiable parameters, see Supplementary Figure S7.

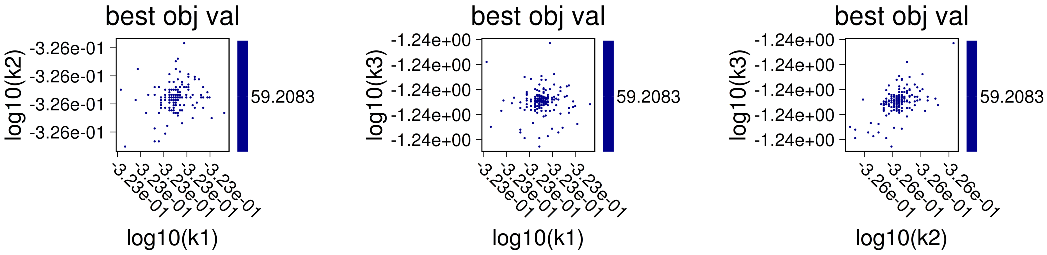

(A)

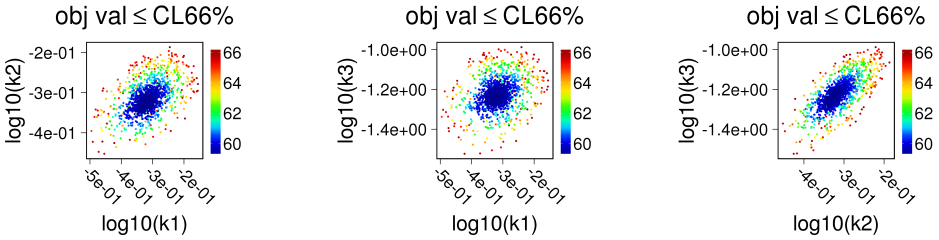

(B)

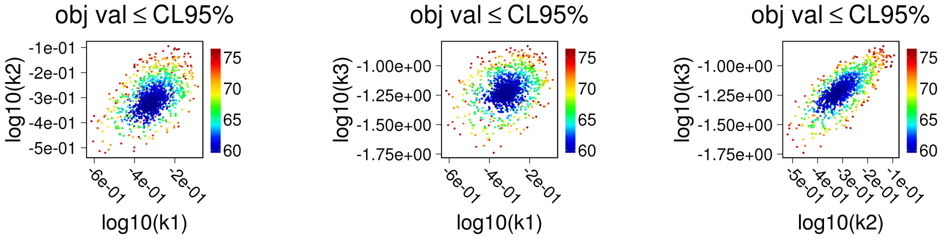

(C)

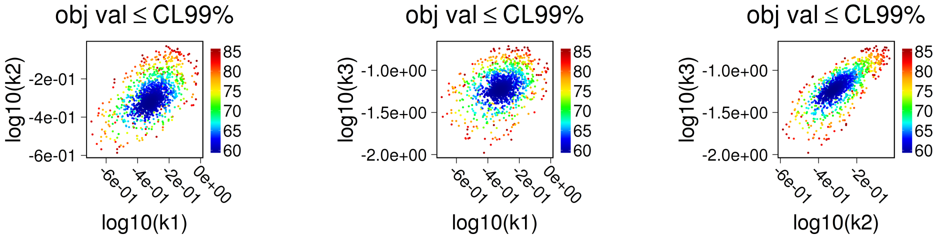

(D)

Figure S5: Parameter estimation pipeline (3rd group). Data generated using the configuration file described in Supplementary Figure S2. This figure shows the correlations between parameters (x/y axes) indicating the fit objective value with the colour (the lower the better). The number of points is determined by the applied filter. The first row only includes the best fits (A), whereas the remaining rows include all the fits within the confidence levels of 66% (B), 95% (C), and 99% (D), respectively, as calculated in Supplementary Figure S3. As in this case the parameters are clearly identifiable, the parameters do not relate with each other. For non-identifiable parameters, see Supplementary Figure S8.

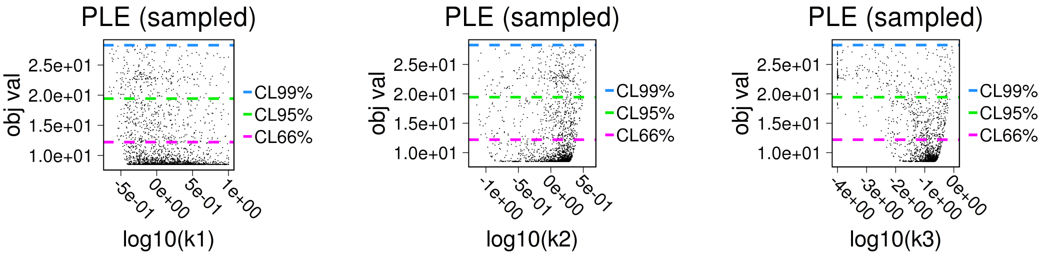

(A)

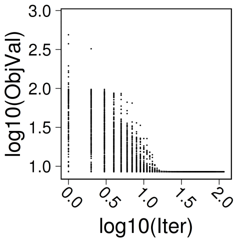

(B)

Figure S6: Parameter estimation pipeline with identifiability issues (1st group). Data generated using the edited version of the configuration file described in Supplementary Figure S2. (A) Using data set shown in Table S2B, the parameters $k1$, $k2$, and $k3$ are not uniquely identifiable. The parameters $k1$ and $k3$ have indefinite right and left confidence intervals respectively. The parameter $k2$ is poorly identified despite the fact that confidence intervals can be estimated. (B) See Supplementary Figure S3 for reference.

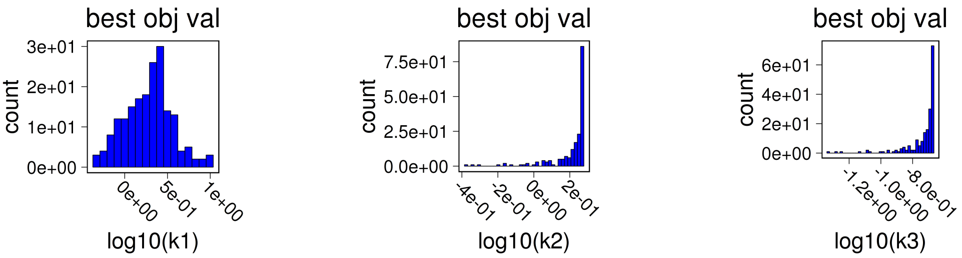

(A)

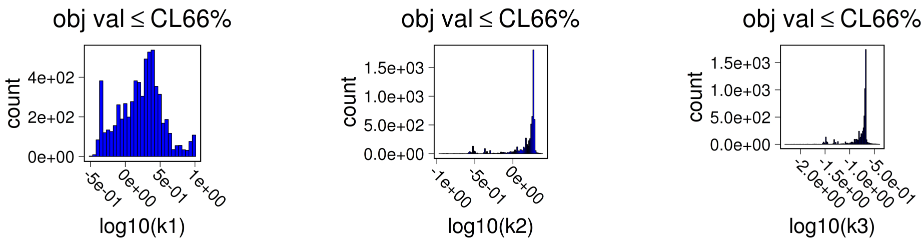

(B)

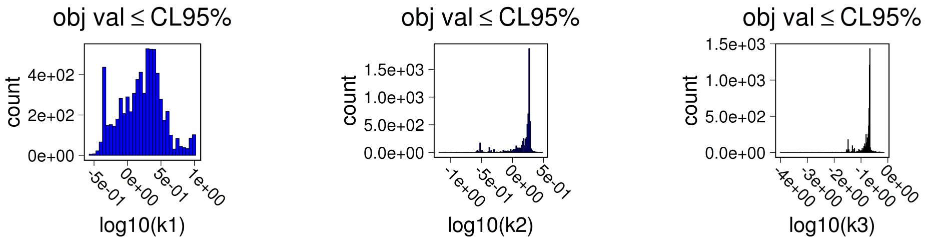

(C)

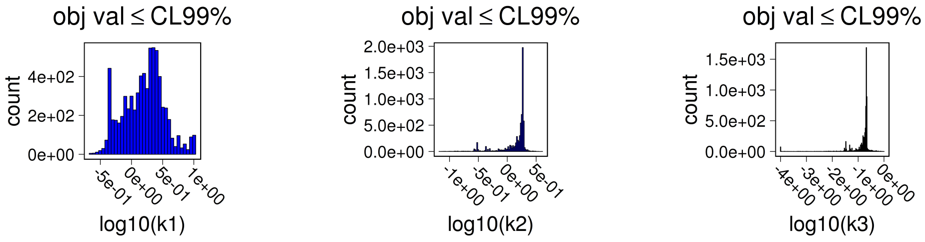

(D)

Figure S7: Parameter estimation pipeline with identifiability issues (2nd group). Data generated using the edited version of the configuration file described in Supplementary Figure S2. Parameter distributions using the best fits (A), all the fits within 66% confidence level (B), 95% confidence level (C), or 99% confidence level (D). The distributions for the parameters $k1$, $k2$, and $k3$ indicate that those parameters are not well defined. In particular $k2$ and $k3$ show skewness and local minima.

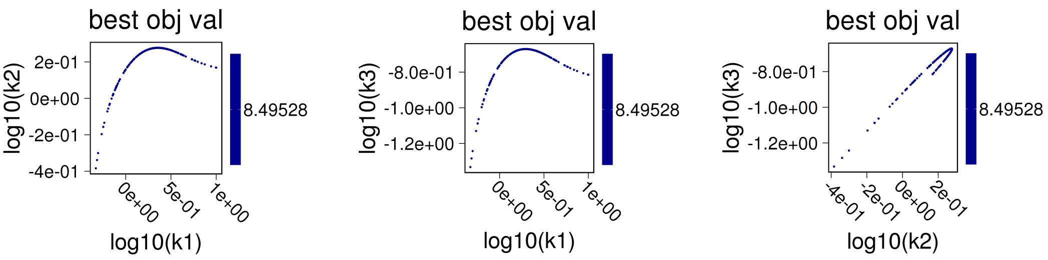

(A)

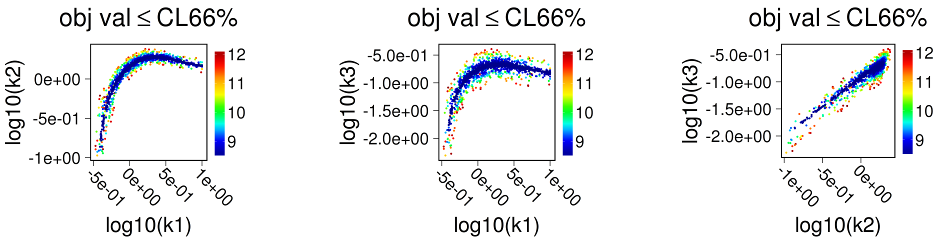

(B)

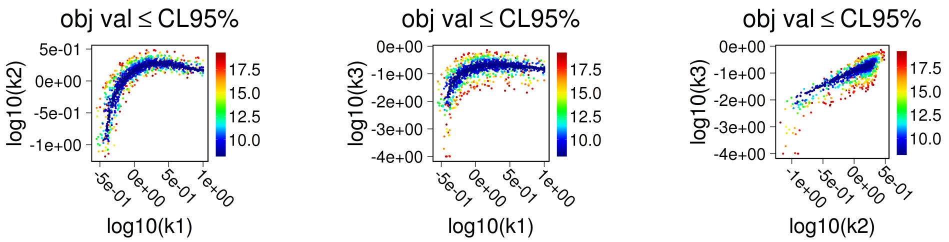

(C)

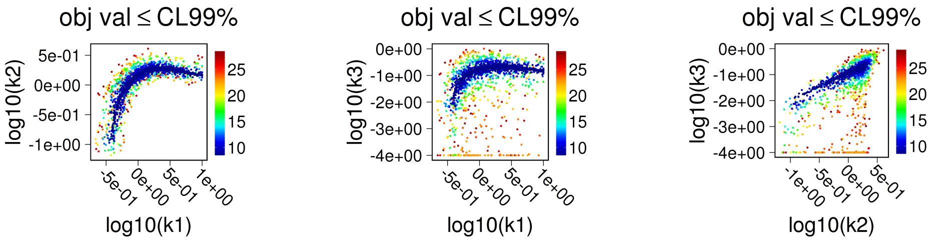

(D)

Figure S8: Parameter estimation pipeline with identifiability issues (3rd group). Data generated using the edited version of the configuration file described in Supplementary Figure S2. Parameter correlations using the best fits (A), all the fits within 66% confidence level (B), 95% confidence level (C), or 99% confidence level (D). The parameters $k1$, $k2$, and $k3$ show non linear correlations in the logarithmic parameter space, particularly due to $k1$.

```
# True if data should be generated, False otherwise
generate_data: True
# True if data should be analysed, False otherwise
analyse_data: True
# True if a report should be generated, False otherwise
generate_report: True
# The relative path to the project directory
project_dir: "."
# The name of the configurator (e.g. Copasi, Rscript, Python, Java)
simulator: "Copasi"
# The model name
model: "insulin_receptor_stoch.cps"
# The cluster type. local if the model is run locally,
# sge/lsf if run on cluster.
cluster: "local"
# The number of CPU if local is used, ignored otherwise
local_cpus: 7
# The number of simulations to perform.
# n>: 1 for stochastic simulations.
runs: 40
# An experimental data set (or blank) to add to the
# simulated plots as additional layer
exp_dataset: "insulin_receptor_dataset.csv"
# True if the experimental data set should be plotted.
plot_exp_dataset: True
# The label for the x axis.
xaxis_label: "Time␣[min]"
# The label for the y axis.
yaxis_label: "Level␣[a.u.]"
```

Figure S9: Example of configuration file for the simulate pipeline. This configuration file was used to generate the Supplementary Figure S10B and the second plot in Figure S10C. A similar configuration file setting:
`model: "insulin_receptor.cps"`
`local_cpus: 1`
`runs: 1`
was used to generate Supplementary Figure S10A and the first plot in Figure S10C. Comments are marked in blue.

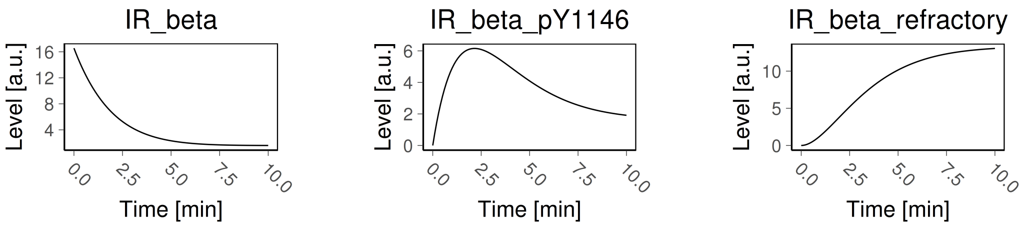

(A)

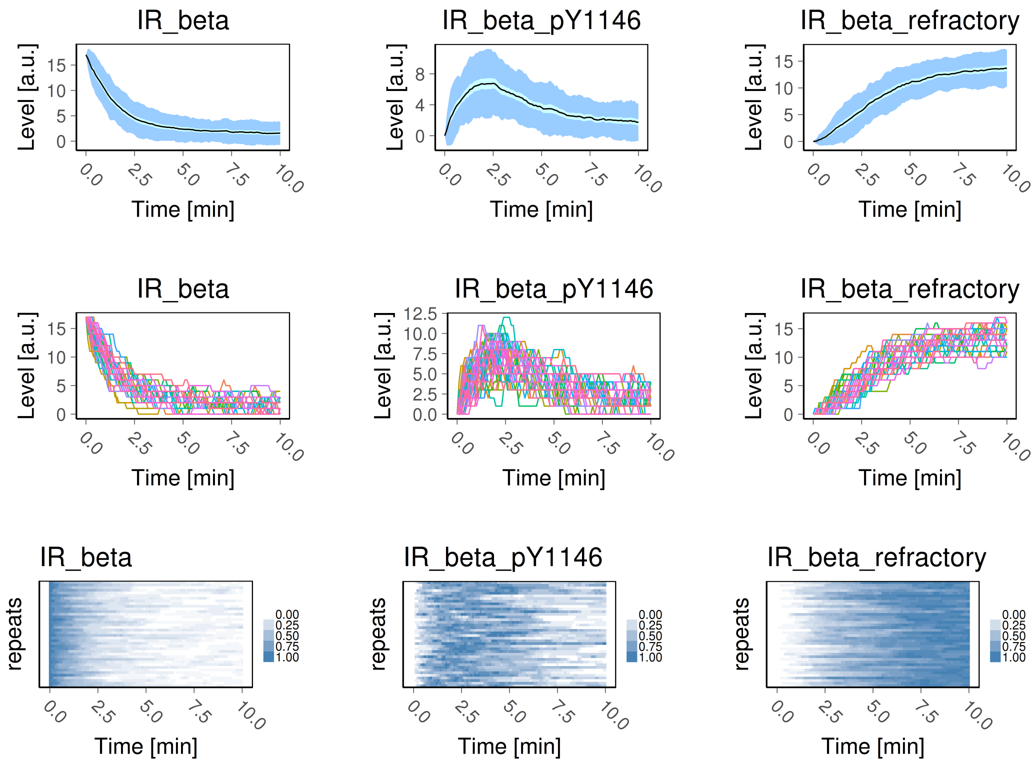

(B)

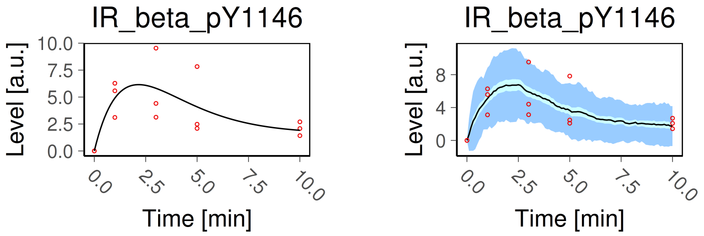

(C)

Figure S10: Simulate pipeline. Data generated using the configuration file described in Supplementary Figure S9. (A) Deterministic simulation using COPASI LSODA algorithm. (B) Stochastic simulation using COPASI Stochastic (Direct method) algorithm. (C) Deterministic and stochastic simulation for the observable variable $IR\beta\_pY1146$ using the first 10 min experimental data from Table S2A. These plots can be disabled using the options `exp_dataset` and `plot_exp_dataset` in the configuration file (see Supplementary Figure S9).

```
# True if data should be generated, False otherwise
generate_data: True
# True if data should be analysed, False otherwise
analyse_data: True
# True if a report should be generated, False otherwise
generate_report: True
# The relative path to the project directory
project_dir: "."
# The name of the configurator (e.g. Copasi)
simulator: "Copasi"
# The model name
model: "insulin_receptor_inhib_scan_IR_beta.cps"
# The variable to scan (as set in Copasi Parameter Scan Task)
scanned_par: "IR_beta"
# The cluster type. local if the model is run locally,
# sge/lsf if run on cluster.
cluster: "local"
# The number of CPU if local is used, ignored otherwise
local_cpus: 7
# The number of simulations to perform per run.
# n>: 1 for stochastic simulations.
runs: 1
# The number of intervals in the simulation
simulate__intervals: 100
# True if the variable is only reduced (knock down), False otherwise.
ps1_knock_down_only: True
# True if the scanning represents percent levels.
ps1_percent_levels: True
# The minimum level (as set in Copasi Parameter Scan Task)
min_level: 0
# The maximum level (as set in Copasi Parameter Scan Task)
max_level: 100
# The number of scans (as set in Copasi Parameter Scan Task)
levels_number: 10
# True if plot lines are the same between scans
# (e.g. full lines, same colour)
homogeneous_lines: False
# The label for the x axis.
xaxis_label: "Time␣[min]"
# The label for the y axis.
yaxis_label: "Level␣[a.u.]"
```

Figure S11: Example of configuration file for the single parameter scan pipeline. This configuration file was used to generate the Supplementary Figure S12A. To generate Supplementary Figure S12B a similar configuration file setting was used:

```
model: "insulin_receptor_k1_scan.cps"
scanned_par: "k1"
ps1_knock_down_only: False
ps1_percent_levels: False
min_level: 0.25119
max_level: 0.843858
levels_number: 100
homogeneous_lines: True
```

Comments are marked in blue.

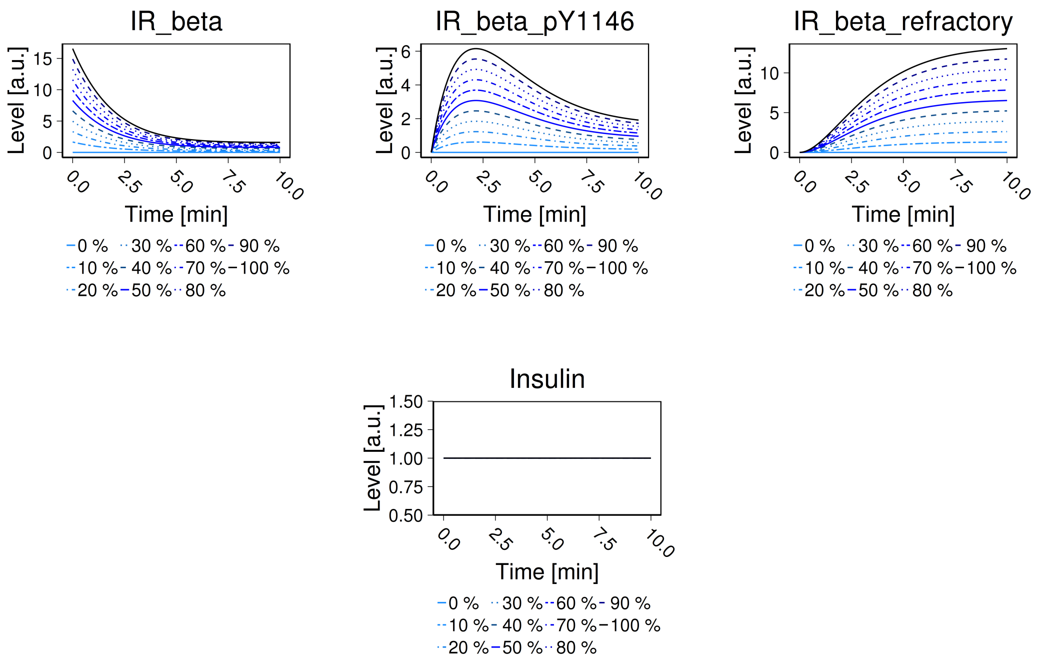

(A)

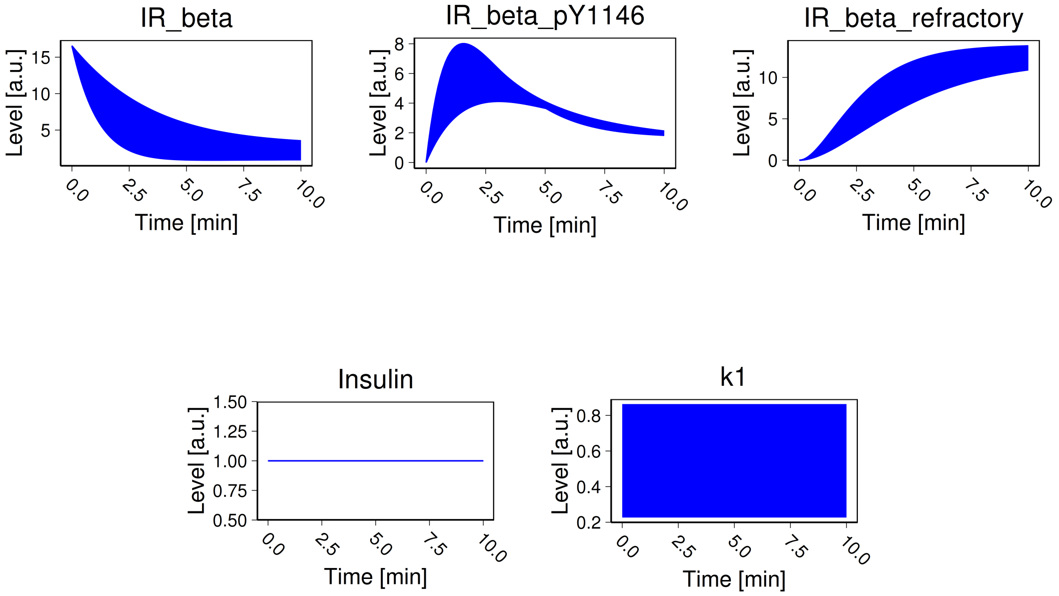

(B)

Figure S12: Single parameter scan pipeline. Data generated using the configuration file described in Supplementary Figure S11. Simulations were generated using LSODA algorithm in COPASI. (A) Parameter scan for $IR\beta Percent$, a species which controls $IR\beta$ using percentages. $IR\beta Percent$ was scanned within the range [0, 100] percent. (B) Parameter scan for $k1$. The parameter $k1$ was scanned within the range [0.25119, 0.843858]. This range corresponds to the confidence interval at 95% confidence level as shown in Supplementary Table S3. Therefore this parameter scan offers an indication of the robustness of the model output upon uncertainty for the parameter $k1$.

```
# True if data should be generated, False otherwise
generate_data: True
# True if data should be analysed, False otherwise
analyse_data: True
# True if a report should be generated, False otherwise
generate_report: True
# The relative path to the project directory
project_dir: "."
# The name of the configurator (e.g. Copasi)
simulator: "Copasi"
# The model name
model: "insulin_receptor_inhib_dbl_scan_InsulinPercent__IRbetaPercent.cps"
# The 1st variable to scan (as set in Copasi Parameter Scan Task)
scanned_par1: "InsulinPercent"
# The 2nd variable to scan (as set in Copasi Parameter Scan Task)
scanned_par2: "IRbetaPercent"
# The cluster type. local if the model is run locally,
# sge/lsf if run on cluster.
cluster: "local"
# The number of CPU if local is used, ignored otherwise
local_cpus: 7
# The number of simulations to perform.
# n>: 1 for stochastic simulations.
runs: 1
# The simulation length (as set in Copasi Time Course Task)
sim_length: 10
```

Figure S13: Example of configuration file for the double parameter scan pipeline. This configuration file was used to generate the Supplementary Figures S14 and S15. Comments are marked in blue.

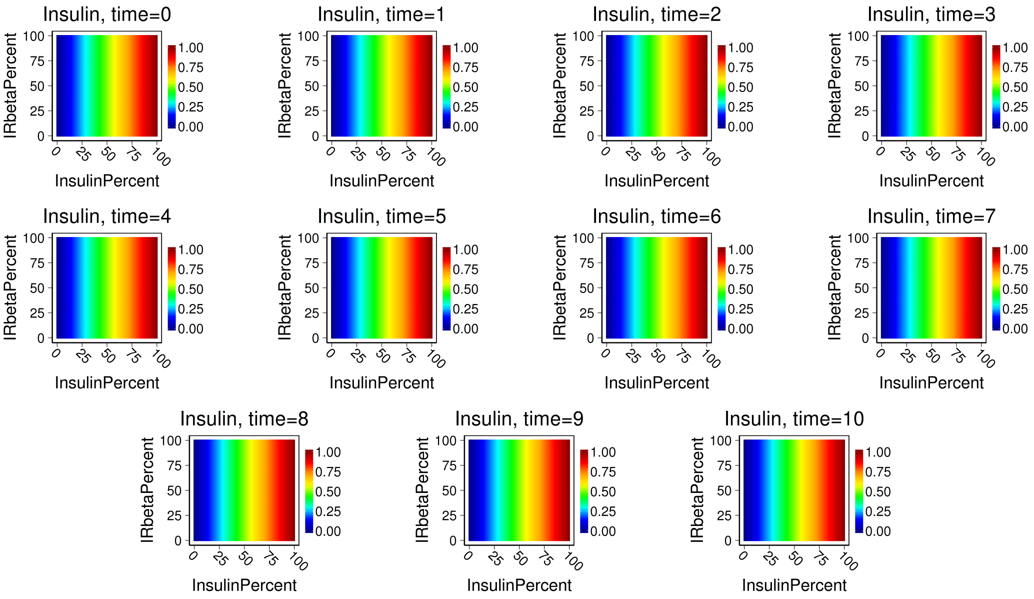

(A)

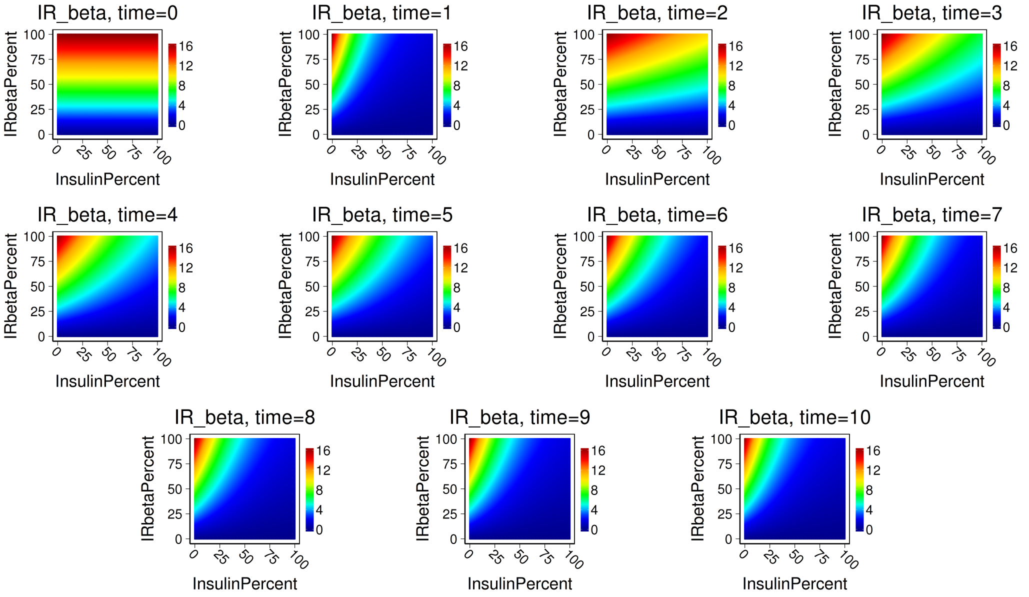

(B)

Figure S14: Double parameter scan pipeline for *Insulin* and $IR\beta$ (1st group). Data generated using the configuration file described in Supplementary Figure S13. Simulations were generated using LSODA algorithm in COPASI. The *InsulinPercent* and $IR\beta Percent$ species were scanned within the range [0, 100] percent. Each plot corresponds to a time point from 0 min to 10 min. Colours indicate species levels in arbitrary units.

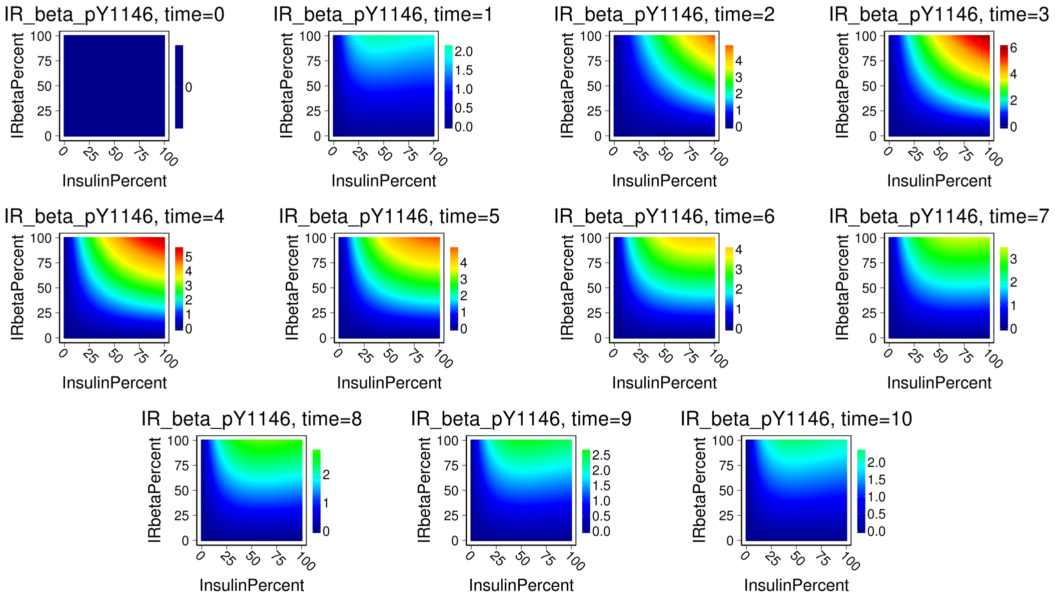

(A)

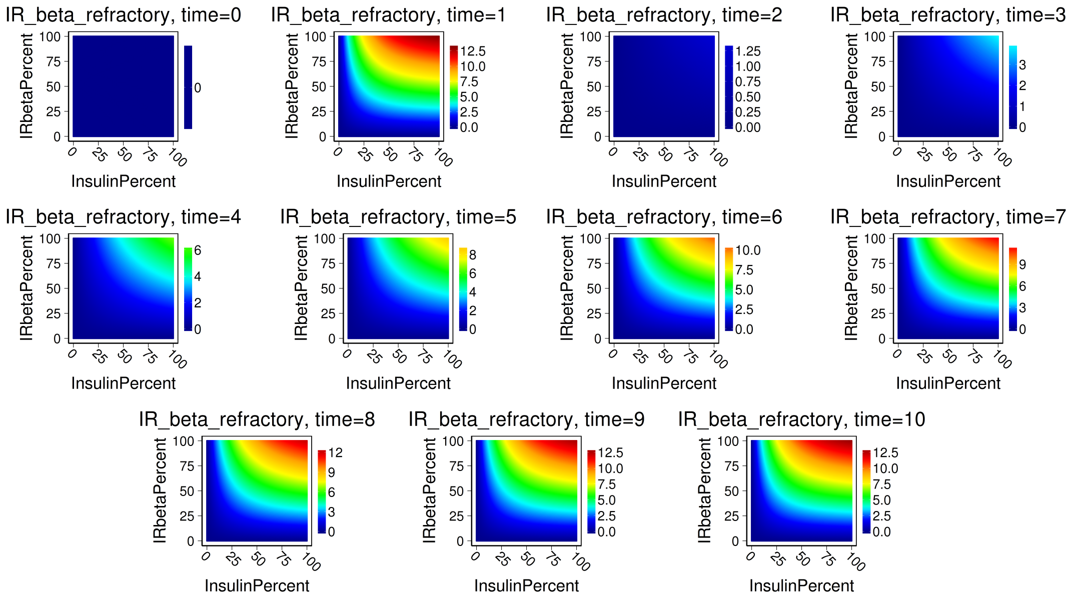

(B)

Figure S15: Double parameter scan pipeline for $IR\beta\_pY1146$ and $IR\beta\_refractory$ (2nd group). Data generated using the configuration file described in Supplementary Figure S13. Simulations were generated using LSODA algorithm in COPASI. The $InsulinPercent$ and $IR\beta Percent$ species were scanned within the range [0, 100] percent. Each plot corresponds to a time point from 0 min to 10 min. Colours indicate species levels in arbitrary units.
